# Substrate binding of human and bacterial type IA topoisomerase: An experimentation with AlphaFold 3.0

**DOI:** 10.1016/j.csbj.2025.03.041

**Published:** 2025-03-27

**Authors:** Yasir Mamun, Ally Aguado, Ana Preza, Abhilasha Kadel, Anjani Mogallur, Briana Gonzalez, Jayleen De La Rosa, Daniel Diaz, Polina Evdokimova, Ukesh Karki, Yuk-Ching Tse-Dinh, Prem Chapagain

**Affiliations:** a Department of Chemistry and Biochemistry, Florida International University, Miami, FL 33199, USA; b Department of Physics, Florida International University, Miami, FL 33199, USA; c Biomolecular Sciences Institute, Florida International University, Miami, FL 33199, USA

**Keywords:** AlphaFold3, Molecular model, Topoisomerase, Substrate binding, Structure prediction, DNA supercoiling, Sequence specificity, Relaxation

## Abstract

Advancements in biophysical techniques such as X-ray crystallography and Cryo-EM have allowed the determination of three-dimensional structures of many proteins and nucleic acids. There, however, is still a lack of 3D structures of proteins that are difficult to crystallize or proteins in complex with other macromolecules. With the advent of deep learning applications such as AlphaFold and RoseTTAFold, it is becoming possible to obtain 3D structures of proteins from their 1D sequences while also generating models of protein-nucleic acid complexes that have been difficult to capture through traditional methods. In this project, we utilized AlphaFold3 (AF3) to create a large number of predicted complexes of two type IA topoisomerases: human topoisomerase 3 beta (hTOP3B) and *Mycobacterium tuberculosis* topoisomerase I bound to a single-stranded DNA (ssDNA). Topoisomerases are enzymes responsible for resolving topological barriers that arise during regular cellular activity. Obtaining structures of topoisomerase complexed with a ssDNA will allow us to discover possible sequence preferences of this enzyme and obtain structures that can be used to screen potential inhibitors. Our analysis showed that AF3 can predict the structure of the enzymes, especially the N-terminal domain, with high confidence. However, predicted protein-DNA complexes, especially with longer (> 25-mer) oligos, are unreliable. The models generated with shorter (9-mer) oligos are obtained with improved confidence and the substrates are placed similarly to crystal structures, but they do not reliably replicate the sequence specificity of the DNA binding of topoisomerase observed in biochemical assays and crystal structures.

## Graphical Abstract

**Figure:**
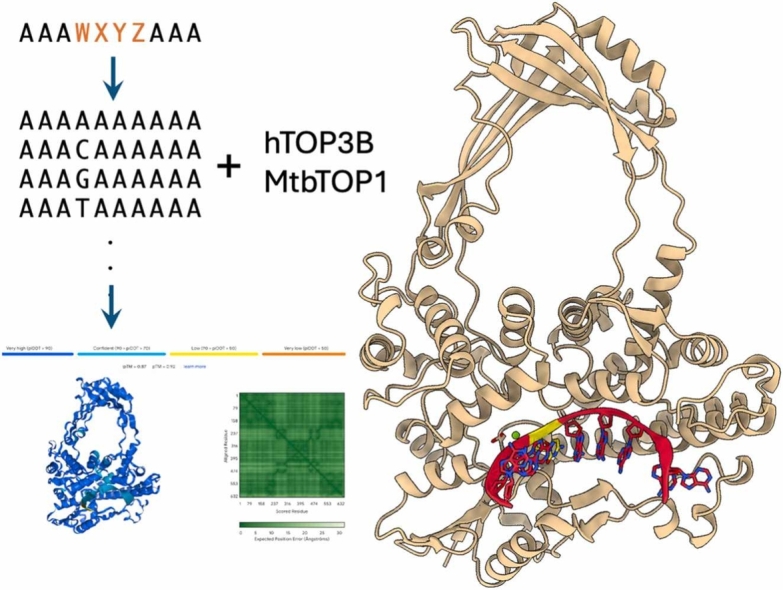

## 1 Introduction

Computationally predicting models of protein and protein complexes is the latest frontier in structural biology, and steady progress has been made in improving the algorithms over the past few decades. The last decade has seen exponential growth in this field, and prediction algorithms based on deep learning, a human neural network-inspired branch of machine learning and artificial intelligence, have significantly changed the landscape [1]. State-of-the-art structure prediction tools such as AlphaFold [2] and RoseTTaFold [3] have recently allowed researchers to predict structures of proteins and biomolecular complexes with unprecedented success. AlphaFold 2, released in 2021, was groundbreaking in predicting 3D protein structure from its 1D sequence with higher accuracy [4]. AlphaFold 2 utilized techniques such as training from the vast amount of deposited 3D structures from the Protein Data Bank (PDB) [5] and learning about residue interactions to form the smaller building blocks of proteins. Since its introduction, it has been extensively used to predict more than 200 million structures of known proteins [6]. However, predicting protein complexes from sequences remained difficult due to the intricate nature of protein interactions with other molecules. Even experimentally, obtaining protein structures in complex with other proteins, nucleic acids, or ligands remains a challenge, as evidenced by the fact that of the more than 200,000 structures deposited in the PDB, only about 13,400 structures are protein-nucleic acid complexes. (https://www.rcsb.org/stats/growth/growth-protein-na-complex). The latest version of AlphaFold, AlphaFold 3 (AF3), is poised to revolutionize the field of predicting protein complexes. It is currently the only tool capable of predicting protein-RNA and protein-antibody complexes [7] with a high degree of accuracy. This has provided an enormous opportunity for researchers to computationally explore structural complexes of biomolecules that were otherwise limited to protein-protein docking tools.

In this study, we utilized the advanced protein-DNA complex prediction capabilities of AF3 to model complexes of DNA substrate bound to bacterial and human type 1 A topoisomerases. Topoisomerases are essential enzymes found in all living organisms, responsible for resolving topological barriers that arise during cellular activities such as transcription, recombination, replication, and DNA repair [8], [9], [10]. Their reaction steps include binding, cleaving, passing, and religating DNA strands to relax supercoiled DNA. Based on the number of DNA strands they impart their activity on, topoisomerases are divided into two classes: type 1 topoisomerases and type 2 topoisomerases. Type 1 topoisomerases work on single-stranded DNA (ssDNA), while type 2 topoisomerases work on double-stranded DNA (dsDNA). Type I topoisomerases are further divided into type IA and type IB. Type IA topoisomerases generally work on negatively supercoiled ssDNA, where they bind with one of the strands of the DNA, known as the G-strand, cut it open, pass the intact strand, known as T-strand, through the cut, and religate the G-strand, thus completing a catalytic cycle [11]. Since supercoiling and relaxation events can occur at any part of a large DNA molecule, topoisomerases generally do not show strict sequence selectivity. Still, continuous work over the years has revealed sequence selectivity in some topoisomerases. Vaccinia virus topoisomerase I shows strong cleavage site preference for the sequence C/TCCTT↑. While several studies have shown that bacterial topoisomerase I, *like E. coli* topoisomerase I (EcTOP1), prefers a cytosine four bases 5′ to the cleavage site, but bacterial topoisomerase III does not [12], [13], [14]. This is possibly due to the cavity formed at the −4 position, that can only accommodate a cytosine [12]. *M. smegmatis* topoisomerase I and *M. tuberculosis* topoisomerase I (MtbTOP1) both recognize and cleave at the sequence C↑TT [14], [15]. In human topoisomerase 3 beta (hTOP3B), another type 1 A topoisomerase, it has been observed that a cytosine five bases 5′ to the cleavage site and a thymine two bases 3′ to the cleavage site is preferred.[16], [17]

To evaluate AF3’s ability to predict DNA substrate sequence selectivity, we screened a large number of DNA sequences against hTOP3B using the AlphaFold server. We first generated models of hTOP3B with a 40-mer oligo used by Yang et al.[17], as well as the fragments generated after cleaving at the sites listed therein and compared the structural basis of substrate binding with the experimental results. To understand if and how AF3 predicts sequence selectivity in the hTOP3B substrate-binding site, we generated hTOP3B-DNA complexes with 9-mer oligos comprising variations in the middle of the sequence (total of 1024 sequences). We repeated the screening of the 9-mer oligos (1024 sequences) against MtbTOP1 and compared the predicted complexes with known cleavage site results and sequence selectivity. Our results show that although the in-silico models generated by AF3 show specific patterns when relative base positions are analyzed, they may not be representative of the experimentally determined activity of the enzyme. Our results provide a basis for comparison when it comes to the prediction of DNA-substrate bound complexes of topoisomerases. Benchmarking various features of the generated models with known observables is important for a more reliable usage of the current abilities and to guide further developments [18], [19].

## 2 Methods

### 2.1 Complex generation

The sequences of hTOP3B and MtbTOP1 were obtained from Uniprot (Uniprot IDs O95985 and P9WG49 respectively) [20]. While we used the full-length sequence of the enzymes for initial predictions, we used the truncated sequences with the same number of residues as in the crystal structures for both proteins to increase the confidence score. For hTOP3B (PDB 5GVC [21]), 611 residues comprising domains D1-D4 were considered, and for MtbTOP1 (PDB 8CZQ [22]), 689 residues comprising domains D1-D5 were considered. These truncated structures exclude the flexible C-terminal domain (CTD). Sequences from Yang et. al [17] were used for the 40-mer oligo along with corresponding fragments (14, 31, and 36-nt). The sequences for the proteins and oligos are listed in Table S1. Shorter 9-mer oligo sequences were used to improve confidence. Since all possible combinations of a 9-mer sequence would be 4^9^ = 262,144, we limited the sequence variations to 1024 by only varying the middle four nucleotides flanked by sequences of adenine (A) or cytosine (C) or thymine (T) or guanine (G). This resulted in sequences with the patterns AAAXXXXAA, CCCXXXXCC, GGGXXXXGG, and TTTXXXXTT (where X represents a variable for A, C, G, or T) with a total of 1024 sequences. The protein sequence, DNA sequence, and an Mg^2 +^ ion were submitted to the AlphaFold server (https://golgi.sandbox.google.com) to predict a TopoI-DNA complex. Jobs were run on the AF3 server and downloaded to local computers for analysis.

### 2.2 Identification of possible cleavage sites

**Fig. 1:**
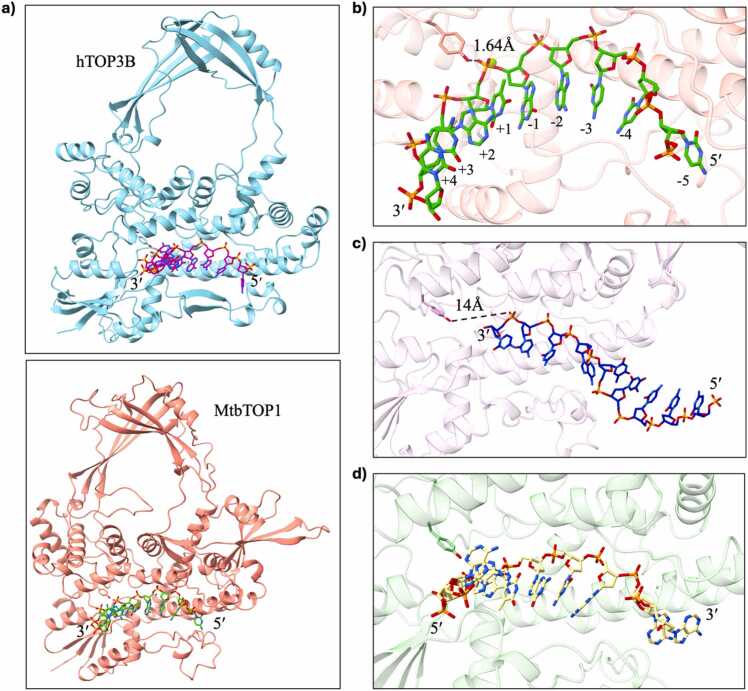
Identification of possible cleavage sites and validation of models. a) Representative AF3 generated models of hTOP3B-DNA complex (top) and MtbTOP1-DNA complex (bottom). b) Identification of the possible cleavage site in a hTOP3B-DNA complex and numbering of base positions relative to the possible cleavage site. c) Representative figure of a hTOP3B-DNA complex where the substrate is placed far away from the binding cavity, making it impossible to identify the possible cleavage site. d) Representative figure of a 5′ and 3′ end flipping observed in some of the hTOP3B-DNA complexes.

In the generated models, the phosphate group that was closest to the catalytic tyrosine was assigned as the possible scissile phosphate and the imminent cleavage site. Python scripts were used to measure the distance between the oxygen atoms of the phosphate groups and the hydroxyl oxygen atom of the catalytic tyrosine and to find the closest phosphate group. The nucleotides towards the 5′ end from this possible cleavage site were numbered −1, −2, and so on, while those towards the 3′ end from the cleavage site were numbered as + 1, + 2, and so on (Figs. 1a, b, S1). The predicted models were checked for accuracy and possible errors. We expected the DNA to be placed in the binding cavity of the N-terminal domain (NTD) of the type IA topoisomerases and the phosphate group of at least one of the nucleotides to be within 4 Å of the catalytic tyrosine in all the best models (model_0). Any substrates that were placed outside or far away from the substrate-binding cavity were excluded from this analysis (Fig. 1c). Also, substrates placed with a flipped orientation, i.e. the 5′ and 3′ ends placed in the opposite direction of what is expected, were excluded (Fig. 1d). After curation, we were left with 990 predicted models of MtbTOP1-DNA and 1024 predicted models of hTOP3B-DNA for further analysis to obtain possible sequence preferences of both type IA topoisomerases.

## 3 Results

### 3.1 Generation of hTOP3B-DNA complex with 40-mer oligo

**Fig. 2:**
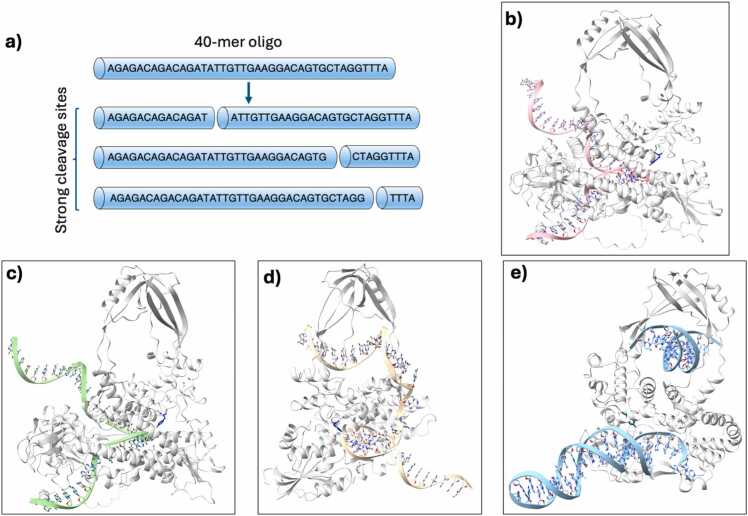
AF3-generated hTOP3B-DNA complex with 40-mer oligo. a) 40-mer oligo and its strong cleavage sites described by Yang et al. [17] that were used for generating the complexes of hTOP3B. b) Representative figure of hTOP3B complexed with 40-mer oligo, with the active site tyrosine (shown in blue, stick formation) near the possible cleavage site of 14-nt. c) Representative figure of hTOP3B complexed with the longer half of the 36-nt cleaved substrate, but the active site tyrosine (shown in blue, stick formation) is positioned closest to the 14-nt. d) In truncated hTOP3B (611 aa), the longer DNA substrates, shown here with the 40-mer oligo, enter the toroid hole. e) This can be prevented by adding a dsDNA, but then the substrate forms a helix with itself.

To determine if AF3 can successfully generate an enzyme-DNA complex, we first decided to generate a complex of hTOP3B with the substrate described in the work of Yang et al. [17] In this work, they use a 40-mer oligo to obtain the possible cleavage activity of hTOP3B and also identify three strong cleavage sites in the oligo at 14, 31, and 36-nt positions (Fig. 2a, Table S1). We used the full-length sequence of hTOP3B (862 aa) with the same 40-mer oligo (sequences in Table S1) as input for AF3. We wanted to compare if AF3 can generate models where the DNA substrate is positioned in a way that mimics the pre-cleavage DNA-bound state and the cleavage site listed in Yang et al. [17], with the base where the cleavage occurs being the closest to the catalytic tyrosine. As previously mentioned, the scissile phosphate closest to the catalytic tyrosine residue was considered the possible cleavage site, and the positions of the bases were analyzed relative to it.

The AF3-modeled protein structure exhibited high confidence (pIDDT>90) across all models except the very end of the C-terminal tail (pIDDT<50), which is flexible but has importance for the T-strand passage [23]. In contrast, the DNA substrate had low confidence (pIDDT<50) in all models except for the bases close to the cleavage site (Figure S2). For the 40-mer oligo, AF3 is found to produce different structures for the protein-DNA complexes in different trials. For example, in two of the five trials we performed, the DNA was modeled with the phosphate group between 14 and 15-nt positioned near the catalytic tyrosine (Fig. 2b), consistent with experimentally observed cleavage sites. However, these models had iPTM scores below 0.55, indicating potentially unsuccessful models. In the other three models, the catalytic tyrosine is close to the site between 5th or 6th nucleotides, but also with low iPTM scores (ranging from 0.59 to 0.64). In all models, the rest of the DNA is modeled as a single helix (Fig. 2b), not interacting with the protein. This is in contrast to the crystal structures, which show that the C terminal of type IA topoisomerases interact with substrates [24], [25]. While some models placed parts of the substrate in the binding cavity, AF3 generally failed to accurately predict the hTOP3B-DNA complex with the 40-mer oligo, producing complexes with consistently low iPTM scores. We then screened shorter oligos by splitting the 40-mer oligo at the known cleavage sites, with the sequences shown in Fig. 2a, resulting in six models. Except for the longer segment of the cleavage at 36-nt, the 5′-end bases of the substrates were placed inside the binding cavity for all the models. The model with 36-nt segment showed a similar binding behavior as the 40-mer oligo (Fig. 2c). Other shorter segments bind within the cavity but with no apparent sequence preference.

We explored whether the confidence scores of the AF3 predictions could be improved by using a truncated enzyme model that included only the sequence corresponding to the crystallographically resolved structure (PDB: 5GVC [21], 611 aa). We found that the iPTM scores for these models improved (>0.70). However, when modeling longer DNA substrates (40-mer, 36-mer, 31-mer), the 3′ tail of the DNA was found to enter the protein's toroid hole in the form of a single helix (Fig. 2d). The toroid hole is considered to accommodate the intact T-strand but not the G-strand bound to the cleavage site, and as such, these AF3-generated models are not likely to be accurate. A recent crystal structure has captured a dsDNA inside the toroid hole of the MtbTOP1 enzyme. [22] To avoid the 40-mer DNA strand from entering the toroid hole, we added a dsDNA so that AF3 could fill the toroid hole. This adjustment allowed AF3 to generate models in which the long single strand (40-mer oligo) does not go through the toroid. However, the structure is predicted as a single-stranded helical structure (Fig. 2e). Overall, we observed that AF3 has a tendency of placing the first few bases of the 5′-end in the binding cavity while extending the rest of the substrate and placing away from the cavity. Based on these findings, we moved on to generate complexes with shorter oligos.

### 3.2 Generation of hTOP3B-DNA complexes with shorter oligos

**Fig. 3:**
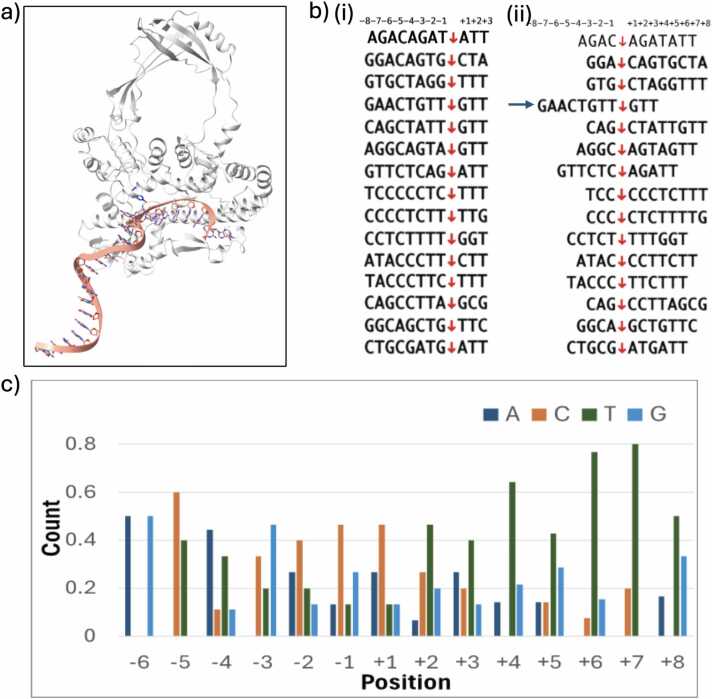
Computationally generated hTOP3B-DNA complex with strong cleavage sites. a) Representative figure of hTOP3B complexed with a 25-mer oligo with the sequence 5′-AGAGACAGACAGATATTGTTGAAGG-3′. b) Comparison of i) the cleavage sites observed by Yang et al. [17] ii) and the possible cleavage site in the models generated by AF3 for 11-mer oligos (Fig. S3 includes the comparison of 15-mer oligos). c) Percent of times a base is observed in the relative position from the cleavage site for the generated hTOP3B-DNA complexes generated with the strong cleavage site sequences, normalized to the total count of bases in each position.

Yang et al. [17] described 15 strong DNA cleavage sites using various 25-mer oligos and found that pyrimidine bases were preferred in several positions, namely C at −5 and T at −2, + 2, and + 3 positions. Since the cleavage occurs at the 14th nucleotide experimentally, we anticipated the catalytic tyrosine to also be near the 14th nucleotide in the AF3-generated models. We observed that the complexes with 25-mer oligos had similar features as the 40-mer oligos, in which the nucleotides in the 5′ end cover the binding cavity while the rest of the sequence forms a single helix. The catalytic tyrosine remains far away from the expected cleavage site (Fig. 3a). Nonetheless, all these models obtained a better iPTM score (> 0.6) than the 40-mer model.

With slight improvements in the iPTM scores for shorter sequences, we decided to use the 11-mer sequences listed in Yang et al. [17]. Each of these shorter oligos includes a cleavage site identified by using 25-mer oligos. The shorter 11-mer oligos are presented so that the cleavage site aligns at the 8th nucleotide from the 5′-end (Fig. 3b i). Nearly all sequences have a C in the −5 position. We generated the complexes with these sequences and analyzed the substrate placement. The predicted positioning was highly variable and did not replicate the possible cleavage site observed in experiments (Fig. 3b ii). Only one sequence (5’-GAACTGTT↓GTT-3’) showed a perfect match in the positioning (Fig. 3b ii). Interestingly, from our aligned data from the AF3-predicted complexes, when we counted instances of the bases in each position, C was observed in the −5 position the highest percentage of times. Similarly, T was observed the highest percentage of times in the + 2 and + 3 positions, but not in the −2 position as expected (Fig. 3c). Interestingly, the positioning changed again when we included an additional four nucleotides, GTTG in the sequences to make 15-mer oligos (Figure S3). An increase in the sequence length only extended the 3’-end out of the cavity but the 5’-end always remained in the cavity, making it highly dependent. Therefore, in general, the models generated by AF3 for the strong cleavage site oligos did not align with the expectations.

### 3.3 Generation of hTOP3B-DNA complexes with 9-mer oligos

**Fig. 4:**
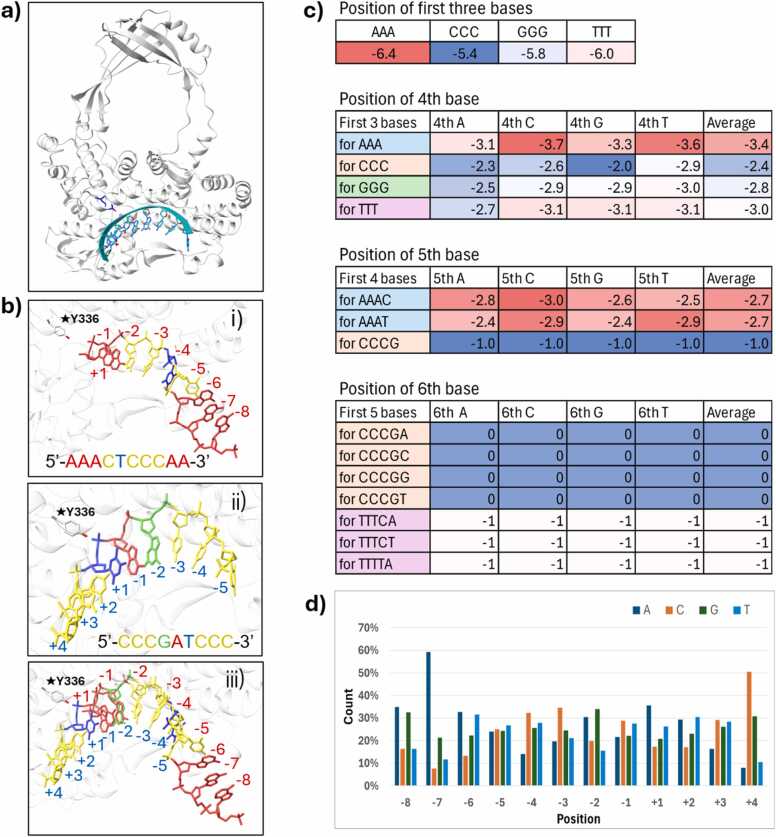
AF3-generated hTOP3B-DNA complex with 9-mer oligo**.** a) Representative figure of modeled hTOP3B complexed with a 9-mer oligo. The substrate (in cyan) is placed inside the binding cavity. Catalytic tyrosine is shown in blue. b) Examples of the numbering of the bases relative to the position of the catalytic TYR336 for the contrasting sequences i) AAACTCCAA and ii) CCCGATCCC. Sequences starting with CCCG- are always placed with first C in the −5 position whereas those starting with AAA- are generally placed with the first few bases outside of the cavity. iii) Superimposition of i) and ii) shows the relative positions for the sequences starting at CCC- vs. AAA- , showing the sequence-dependent position shift in the predicted complexes. c) Table illustrating the shifting of the base positions depending on the first/previous bases for the substrates of the predicted hTOP3B-9-mer DNA complexes for selected sequences. d) Percent of times a base in observed in the relative position from the cleavage site for the predicted hTOP3B-9-mer DNA complexes, normalized to the total count of bases in each position.

As longer oligos did not produce reliable results, we attempted to generate models with shorter oligos to see if the accuracy could be improved. For this, we used 9-mer oligo sequences to generate the complexes. With all combinations, there would be 4 [9] = 262,144 9-mer sequences. To limit the number of screens, we made only the middle four nucleotides as variables and left the three nucleotides at either end as all As, all Cs, all Gs, or all Ts. For example, the 9-mer sequence with As in either end would be AAAXXXXAA, with 256 exhaustive combinations of XXXX. Using these sequences, we generated 1024 models. A representative hTOP3B in complex with a 9-mer substrate is shown in Fig. 4a. All generated complexes had the substrate placed in the cavity and properly oriented. When aligned along the possible cleavage site, the 9-mer oligo structures range from the positions −8 to + 3. We counted the number of times a base lies in a specific position and plotted (as a percentage) in Fig. 4d, which shows that A has a strong preference for −7 position, and C has a slight preference for the −3 and −4 positions.

To identify any sequence preference in AF3 predictions, we next analyzed how the sequences starting with AAA or CCC or GGG or TTT were placed. We observe that the first three repeat bases can influence the starting position of the substrate in the AF3-generated models. For the sequences with the first three bases as AAA, the preferred starting position was either −7 or −6. When averaged over the positions of all sequences starting at AAA, we get −6.4 (Fig. 4b,c). When the sequences beginning with AAA are placed starting at the −8 position, the first two nucleotides apparently overhang outside of the cavity as shown in Fig. 4b i). In contrast, for the sequences with the first three bases as CCC, the preferred starting position was −5 (with an average of −5.4) and for the sequences with GGG and TTT, the preferred starting position was −6. We then examined the effects of the fourth base on the sequence placement. If the fourth base is either C or T after AAA, the fourth base is most likely to be placed in the −4 position, and the substrate starts from the −7 position. On the other hand, if the first three bases are CCC and the fourth base is G, the fourth base was always placed in the −2 position, with the substrate starting from the −5 position. The substrate placement for a sequence starting in CCCG- is shown in Fig. 4b (ii) and Figure S4a. This contrasts with the sequence CCCT, where the fourth base T was almost always in the −3 position, i.e. the first C in −6. This is displayed in Figure S4b which shows one nucleotide shift in the placement of the sequence starting in CCCT- with the first C placed at −6. Shift analysis with the fifth and sixth base in the sequence showed that, if the first four bases are AAAC and AAAT, the next base would be placed preferentially in the −3 position. Interestingly, for the first bases as CCCG, regardless of what the fifth base is, its position was always −1. Indeed, this is clearly seen in the table in 4c, which shows that the sixth base is in the 0 position for any combination of CCCGX, where X is either A, C, G, or T. As shown in Figure S4a, multiple sequences starting in CCCG- overlap quite well with the first C placed at −5 position. Position 0 is also the site where the covalent bond forms and this site has traditionally been numbered + 1 (i.e. the base position in literature is numbered as …, −3, −2, −1, +1, +2, +3, …). While the starting sequence CCC showed a strong position preference in these AF3 predictions, sequences starting in GGG and TTT did not show clear patterns, except that the preferred starting position was −6. Overall, the AF-predicted models show specific bias in the substrate placement (e.g. first C placed at –6 for the sequences starting with CCCG-), but did not yield the expected base preferences for C in the −5 position.

### 3.4 Substrate placement in MtbTOP1 for 9-mer oligos

With the 9-mer oligo binding to hTOP3B showing sequence-dependent variability in AF3 predicted structures as discussed above, we further tested the substrate placement predictions for MtbTOP1, which is also a type IA topoisomerase but one of the handful of topoisomerases that show sequence selectivity. [26]

**Fig. 5:**
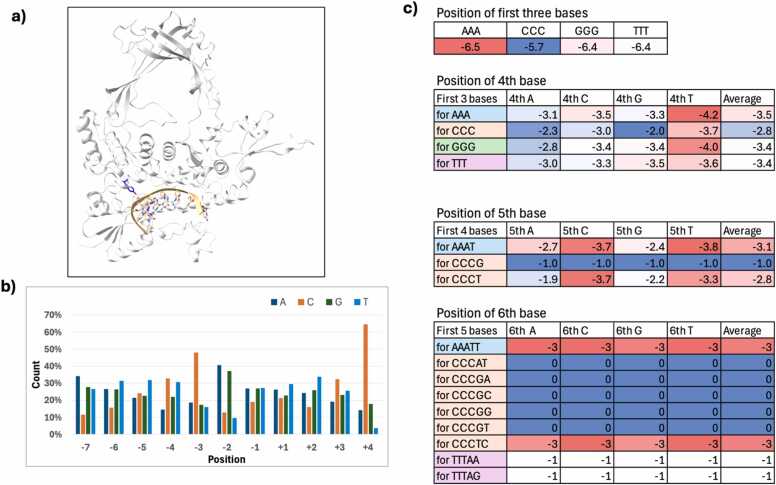
AF3-generated MtbTOP1-DNA complex with 9-mer oligo. a) Representative figure of the modeled MtbTOP1, complexed with a 9-mer oligo. The substrate (in yellow) is placed inside the binding cavity. Catalytic tyrosine is shown in blue. b) Percent of times a base is observed in the relative position from the cleavage site for the modeled MtbTOP1–9-mer DNA complexes, normalized to the total count of bases in each position. c) Table illustrating the shifting of the base positions depending on the first/previous bases for the substrates of modeled MtbTOP1–9-mer DNA complexes for selected sequences.

The prototypical type IA enzyme EcTOP1 shows a preference for cytosine in the −4 position, with its crystal structure (PDB ID 3PX7) [12] providing the structural basis for the preference of a C at −4. MtbTOP1 exhibits a strong cleavage site pattern of C↑TT while also preferring a C at −4 position relative to the cleavage site [15]. To determine whether AF3 can replicate these observations, we analyzed complexes generated with the 9-mer oligos. If that were the case, we would expect C to occur most frequently at −4, while C, T, and T would occur predominantly at positions −1, + 1, and + 2, respectively. In most of AF3-generated MtbTOP1-DNA models, the substrate was placed within the binding cavity (Fig. 5a). However, the models lacked a clear preference for cytosine at −4 position from the cleavage site. Instead, when all the models are considered, C is found to be preferentially placed at −3 or + 4 positions whereas A and G are more common at −2 (Fig. 5b). Analysis of the 9-mer oligo substrate placement in MtbTOP1 shows distinct positional patterns (Fig. 5c). If the first three bases are CCC, the preferred starting position is mostly between −5 and −6, whereas it is between −6 and −7 for other starting triplicates. The observation that the starting base C is always placed in the −5 position for CCCG is the same as in hTOP3B. This seems to be a clear modeling preference or bias of AF3. For sequence starting in CCCG, the 4th base G is placed at −2, but when G is changed to T, i.e. for sequence CCCT, the 4th base T is placed at −3, although they both have the same first three bases CCC. Extending our calculations for up to the 6th base, we observed that for all sequences starting with CCCGXX, the first C is placed at −5, where XX could be any base combination. Similarly, if the first four bases are AAAT, the preferred position of the fifth base is −3. Overall, these results show variability in base positioning across sequences, highlighting context-dependent modeling tendencies in AF3's predictions.

Recent Cryo-EM [27] and MD simulation [28] data have revealed the interacting residues near the ssDNA for hTOP3B. Similarly, ssDNA interactions with nearby residues have also been established for MtbTOP1 through X-ray crystallography. [22], [25] To understand if the interactions between the nucleotides and nearby residues influence the base position specificity in the predicted models, we chose two sequences, 5’-CCCGATCC-3’ (from −5 to +4) and 5’-AAACTCCAA-3’ (from −8 to +1), for both hTOP3B and MtbTOP1, and analyzed the interactions (Figure S5). In our predicted models of hTOP3B, we also observed the same residues interacting with the DNA backbone (R524, S513, T510, R338, etc.) or the bases (R194, H60, etc.) as expected [27]. It has been established that a C is preferred in the −5 position [17] and R194 may be responsible for the specificity. In both of our predicted complexes, C is placed in the −5 position. However, R194 is found to interact with C at −5 in one sequence but with T in −4 position for the other. Similarly, experiments show that a C is preferred in the −4 position in MtbTOP1 [15]. While the interacting residues are the same in the generated models (Figure S5), C is found in either −4 or −3 position, lacking a clear specificity.

## 4 Conclusion

In this study, we analyzed the AlphaFold 3 (AF3)-modeled complexes of type IA topoisomerase-DNA to assess whether the DNA substrate positioning aligns with the experimental observations. As protein-nucleic acid complexes are difficult to obtain, we explored the fidelity of the AF3-generated complexes in the context of nucleotide sequence preference. We find that while AF3 can correctly place short oligonucleotide substrates within the binding cavity, it fails to accurately reproduce sequence-dependent substrate positioning in type IA topoisomerase-DNA complexes. Even when the DNA substrates are placed near the catalytic tyrosine (i.e. putative catalytic site), the predicted model complexes show low confidence scores. This is possibly because only a handful of DNA-bound type-1A topoisomerases exist in the protein databank (e.g. 3PX7, 4RUL, 1I7D, 6K8O, 2O5C, 2O5E, 6CQ2, 2O54, 2O59, 2O19). Additionally, the DNA fragments in the complexes are quite short (typically 7 nt) and the sequence variation is not exhaustive. From the sequences that we used for our modeling exercise, we observed specific patterns for positioning particular sequences. For example, sequences starting with CCC- are preferentially placed with first C in the –5 or −6 position whereas those starting with AAA- are generally placed with the first A shifted to the –6 or –7 position. For both hTOP3B and MtbTOP1, the first C was always placed in –5 position for any sequence starting with CCCG-. However, these patterns likely represent modeling artifacts rather than actual structural features of the complexes, considering the lack of reliable predictions of known base-preferences (e.g. C in –5 position in hTOP3B, and C in –4 preference in EcTOP1). Despite the lack of the known base specificity, the neighboring amino acid residues of the enzymes interacting with the substrate generally matched well in the AF-predicted models of both hTOP3B and MtbTOP1. Notably, R194 interaction is considered important for base specificity of C at −5 position [17] for hTOP3B, but the models show R194 interactions with nucleotide either at –4 or –5, even for sequences that correctly place C at –5. Also, while the interacting residues [15] are the same in the AF-generated models of MtbTOP1, C is found in either −4 or −3 position, instead of known –4 position [15].

Generative artificial intelligence algorithms like AF3 remain powerful and invaluable tools for understanding the structure and biochemistry of biomolecules, significantly advancing our ability to model both natural and engineered complexes. While there will be continuous improvements in the prediction algorithms, benchmarking various features of the generated models with known observables is important for a more reliable usage of the current abilities and to guide further developments.

## CRediT authorship contribution statement

**Mamun Yasir:** Writing – review & editing, Writing – original draft, Visualization, Validation, Supervision, Methodology, Investigation, Formal analysis. **Aguado Ally:** Validation, Investigation, Data curation. **Preza Ana:** Validation, Investigation, Data curation. **Kadel Abhilasha:** Validation, Investigation, Data curation. **Mogallur Anjani:** Validation, Investigation, Data curation. **Gonzalez Briana:** Validation, Investigation, Data curation. **De La Rosa Jayleen:** Validation, Investigation, Data curation. **Diaz Daniel:** Validation, Investigation, Data curation. **Evdokimova Polina:** Validation, Investigation, Data curation. **Karki Ukesh:** Software, Investigation. **Tse-Dinh Yuk-Ching:** Writing – review & editing, Validation, Supervision, Investigation, Funding acquisition. **Chapagain Prem P.:** Writing – review & editing, Validation, Supervision, Resources, Project administration, Methodology, Investigation, Formal analysis, Data curation, Conceptualization.

## Declaration of Competing Interest

No conflicts of interest to declare.
