# Supplementary material for: Substrate binding of human and bacterial type IA topoisomerase: An experimentation with AlphaFold 3.0
Source: Comput Struct Biotechnol J. 2025 Mar 27;27:1342–9. doi: 10.1016/j.csbj.2025.03.041 (PMC11999072; doi:10.1016/j.csbj.2025.03.041)
Supplement: Supplementary file 1 — Supplementary material [file mmc1.docx]

**Supplemental Information for:**

**Substrate binding of human and bacterial type IA topoisomerase: An experimentation with AlphaFold 3.0**

Table 1: Sequences used for generating models

| Human topoisomerase 3 beta | MKTVLMVAEKPSLAQSIAKILSRGSLSSHKGLNGACSVHEYTGTFAGQPVRFKMTSVCGHVMTLDFLGKYNKWDKVDPAELFSQAPTEKKEANPKLNMVKFLQVEGRGCDYIVLWLDCDKEGENICFEVLDAVLPVMNKAHGGEKTVFRARFSSITDTDICNAMACLGEPDHNEALSVDARQELDLRIGCAFTRFQTKYFQGKYGDLDSSLISFGPCQTPTLGFCVERHDKIQSFKPETYWVLQAKVNTDKDRSLLLDWDRVRVFDREIAQMFLNMTKLEKEAQVEATSRKEKAKQRPLALNTVEMLRVASSSLGMGPQHAMQTAERLYTQGYISYPRTETTHYPENFDLKGSLRQQANHPYWADTVKRLLAEGINRPRKGHDAGDHPPITPMKSATEAELGGDAWRLYEYITRHFIATVSHDCKYLQSTISFRIGPELFTCSGKTVLSPGFTEVMPWQSVPLEESLPTCQRGDAFPVGEVKMLEKQTNPPDYLTEAELITLMEKHGIGTDASIPVHINNICQRNYVTVESGRRLKPTNLGIVLVHGYYKIDAELVLPTIRSAVEKQLNLIAQGKADYRQVLGHTLDVFKRKFHYFVDSIAGMDELMEVSF |
| --- | --- |
| *M. Tuberculosis* topoisomerase 1 | SGRRLVIVESPTKARKLASYLGSGYIVESSRGHIRDLPRAASDVPAKYKSQPWARLGVNVDADFEPLYIISPEKRSTVSELRGLLKDVDELYLATDGDREGEAIAWHLLETLKPRIPVKRMVFHEITEPAIRAAAEHPRDLDIDLVDAQETRRILDRLYGYEVSPVLWKKVAPKLSAGRVQSVATRIIVARERDRMAFRSAAYWDILAKLDASVSDPDAAPPTFSARLTAVAGRRVATGRDFDSLGTLRKGDEVIVLDEGSATALAAGLDGTQLTVASAEEKPYARRPYPPFMTSTLQQEASRKLRFSAERTMSIAQRLYENGYITYMRTDSTTLSESAINAARTQARQLYGDEYVAPAPRQYTRKVKNAQEAHEAIRPAGETFATPDAVRRELDGPNIDDFRLYELIWQRTVASQMADARGMTLSLRITGMSGHQEVVFSATGRTLTFPGFLKAYVETVDELVGGEADDAERRLPHLTPGQRLDIVELTPDGHATNPPARYTEASLVKALEELGIGRPSTYSSIIKTIQDRGYVHKKGSALVPSWVAFAVTGLLEQHFGRLVDYDFTAAMEDELDEIAAGNERRTNWLNNFYFGGDHGVPDSVARSGGLKKLVGINLEGIDAREVNSIKLFDDTHGRPIYVRVGKNGPYLERLVAGDTGEPTPQRANLSDSITPDELTLQVAEELFAT |
| 40-mer sequence from Yang et al.^1^ | AGAGACAGACAGAT↑ATTGTTGAAGGACAGTG↑CTAGG↑TTTA |
| 11-mer dsDNA sequence | CTTCCGCTTGA  GAAGGCGAACT |
| 25-mer sequences from Yang et al.^1^ | AGAGACAGACAGAT↑ATTGTTGAAGG |
|  | AGAGACGGACAGTG↑CTAGTTGAAGG |
|  | AGAGACGTGCTAGG↑TTTGTTGAAGG |
|  | AGAGACGAACTGTT↑GTTGTTGAAGG |
|  | AGAGACCAGCTATT↑GTTGTTGAAGG |
|  | AGAGACAGGCAGTA↑GTTGTTGAAGG |
|  | AGAGACGTTCTCAG↑ATTGTTGAAGG |
|  | AGAGACTCCCCCTC↑TTTGTTGAAGG |
|  | AGAGACCCCCTCTT↑TTGGTTGAAGG |
|  | AGAGACCCTCTTTT↑GGTGTTGAAGG |
|  | AGAGACATACCCTT↑CTTGTTGAAGG |
|  | AGAGACTACCCTTC↑TTTGTTGAAGG |
|  | AGAGACCAGCCTTA↑GCGGTTGAAGG |
|  | AGAGACGGCAGCTG↑TTCGTTGAAGG |
|  | AGAGACCTGCGATG↑ATTGTTGAAGG |
| 11-mer sequences from Yang et al.^1^  (15-mer sequences included additional four bases in the 3’ end GTTG). | AGACAGATATT |
|  | GGACAGTGCTA |
|  | GTGCTAGGTTT |
|  | GAACTGTTGTT |
|  | CAGCTATTGTT |
|  | AGGCAGTAGTT |
|  | GTTCTCAGATT |
|  | TCCCCCTCTTT |
|  | CCCCTCTTTTG |
|  | CCTCTTTTGGT |
|  | ATACCCTTCTT |
|  | TACCCTTCTTT |
|  | CAGCCTTAGCG |
|  | GGCAGCTGTTC |
|  | CTGCGATGATT |
| 9-mer sequences (this work) | AAAXXXXAA |
|  | CCCXXXXCC |
|  | GGGXXXXGG |
|  | TTTXXXXTT |

* ↑ represents the cleavage sites. X= A, C, G, T


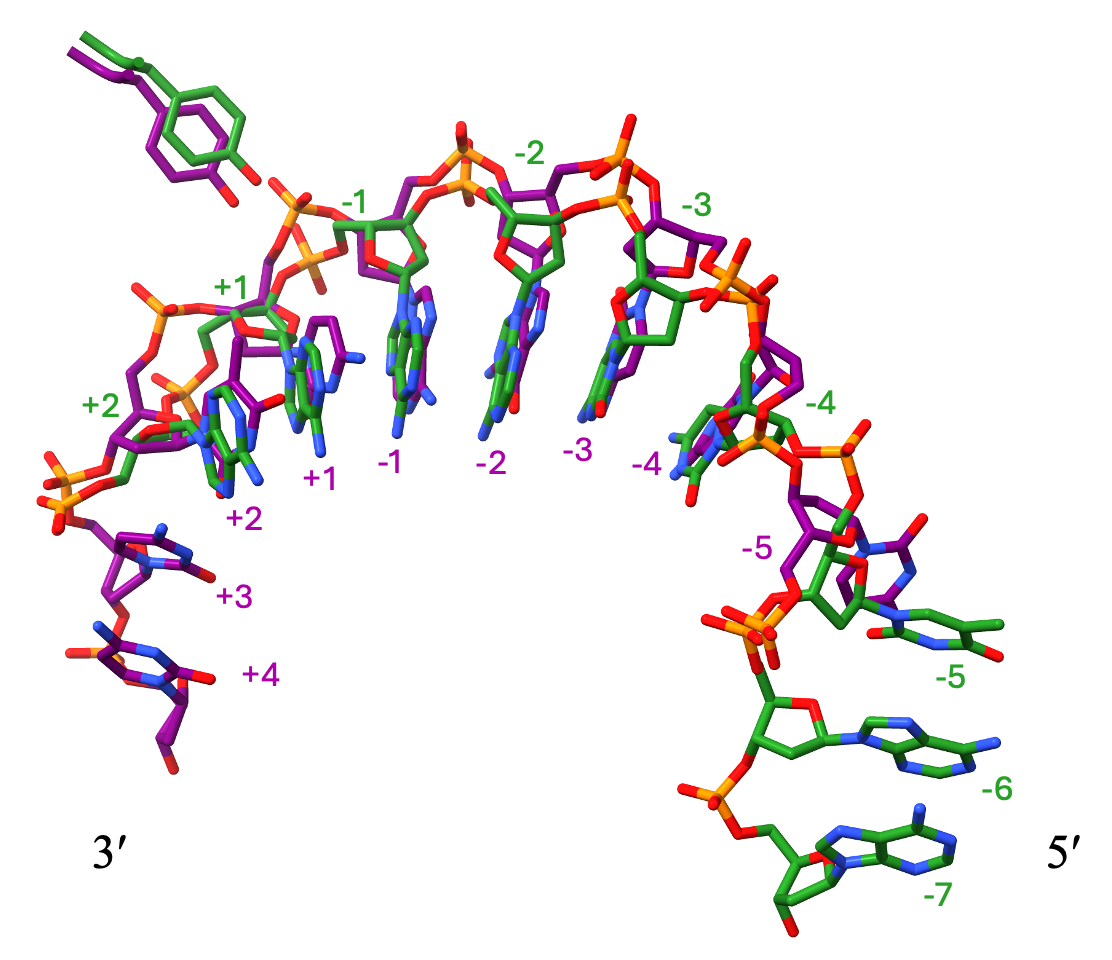


Figure S1: Example of the numbering of base positions relative to catalytic tyrosine shown by superimposing two different models from the MtbTOP1-DNA complex


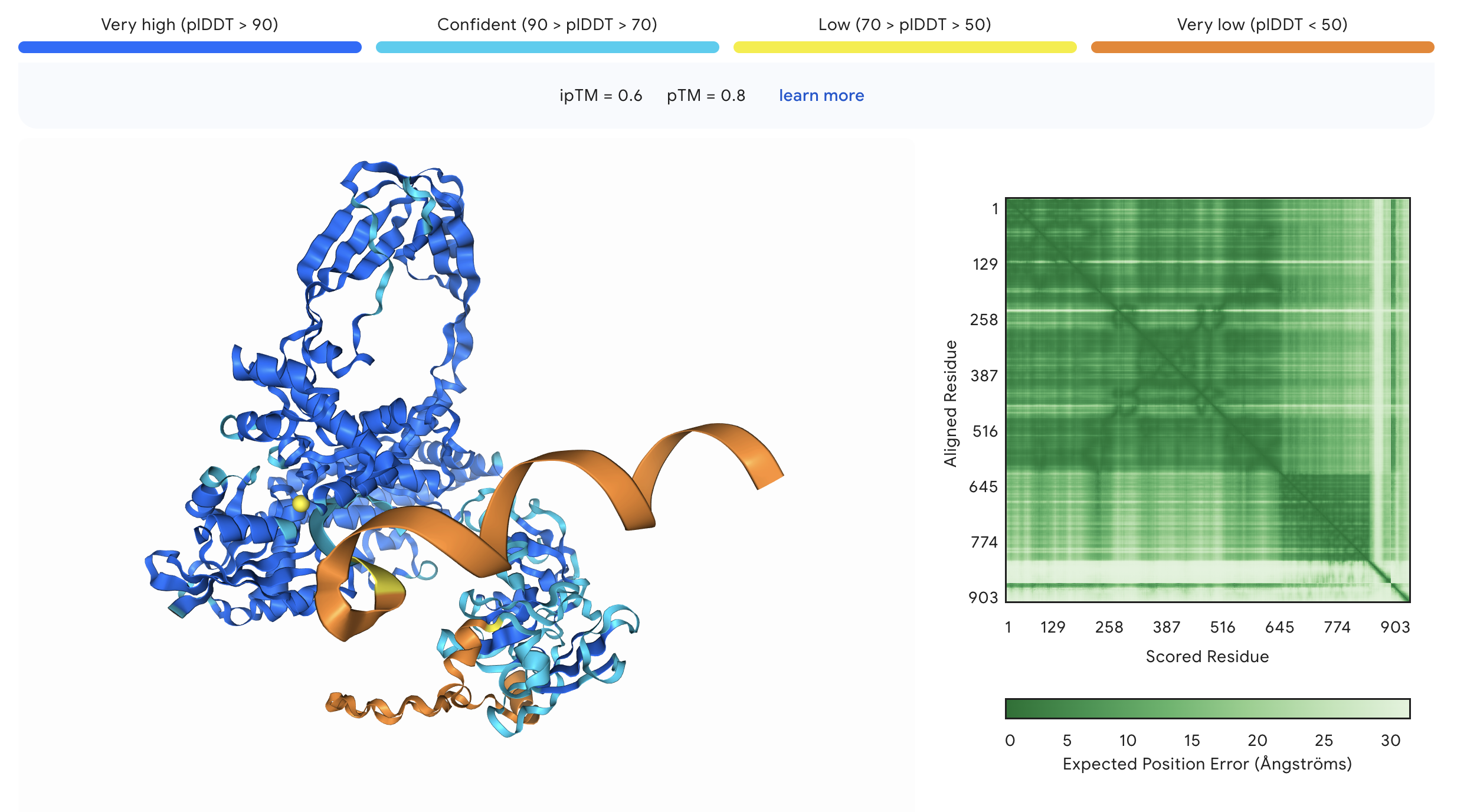


Figure S2: Screenshot showing the confidence score of different regions and the overall model of the hTOP3b-DNA complex with 40-mer oligo. The protein structure has a high confidence score other than the end of the C-terminal tail, while the DNA shows good confidence only for the bases towards the 5ʹ end inside the binding cavity. The iPTM score for the model is also low (0.60), indicating this prediction was probably unsuccessful.


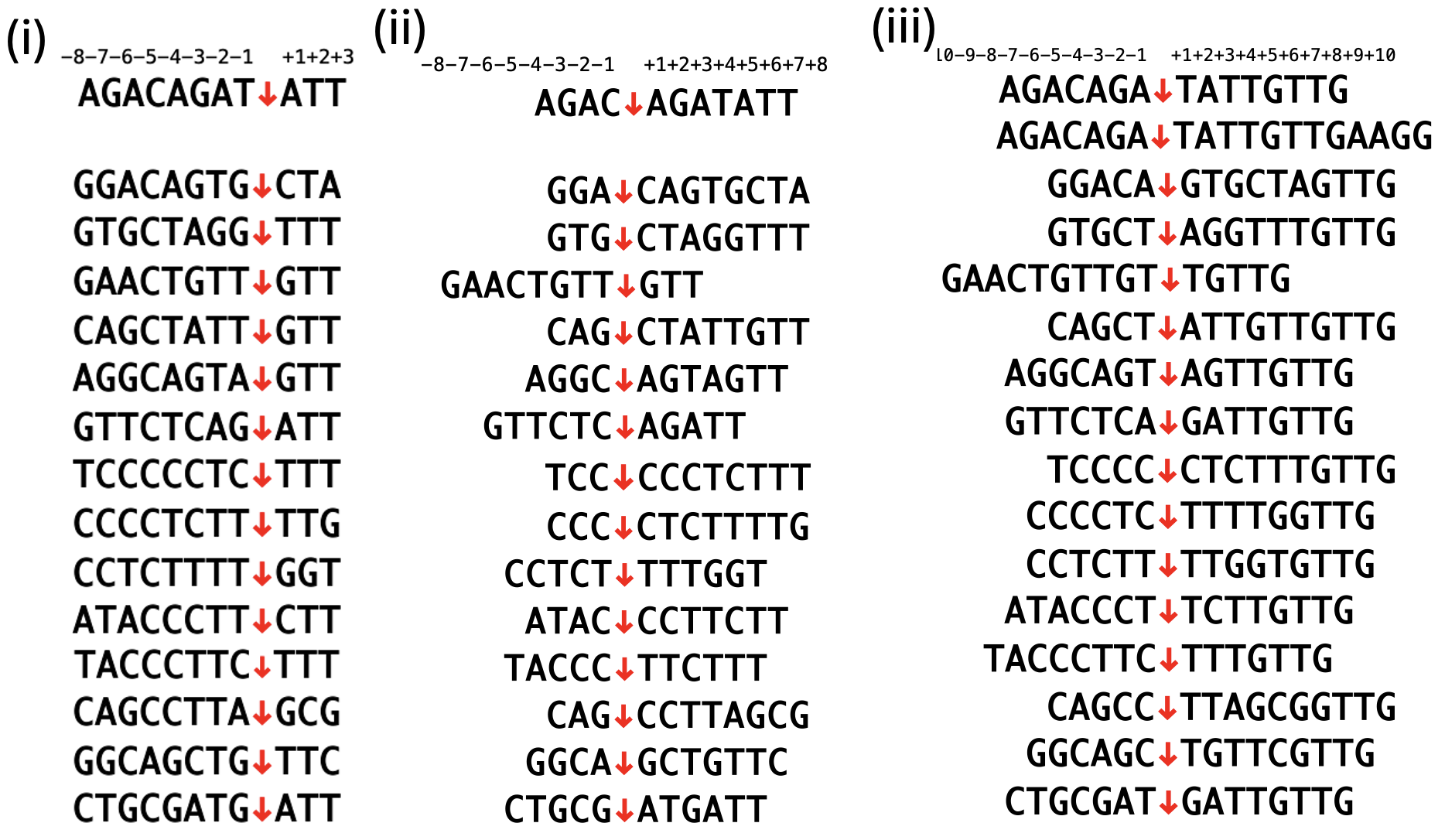


Figure S3. Comparison of the experimental and AF3-predicted cleavage sites. (i) The cleavage sites identified in Yang et al.^1^ ii) AF3-predicted sites for the same 11-mer sequences as in (i). iii) When the sequence length is increased, the starting position generally shifts leftward so that the cleavage site sifts towards 3’ end. Further increase in the sequence length appears to only extend the 3’ end structure protruding out of the cavity.


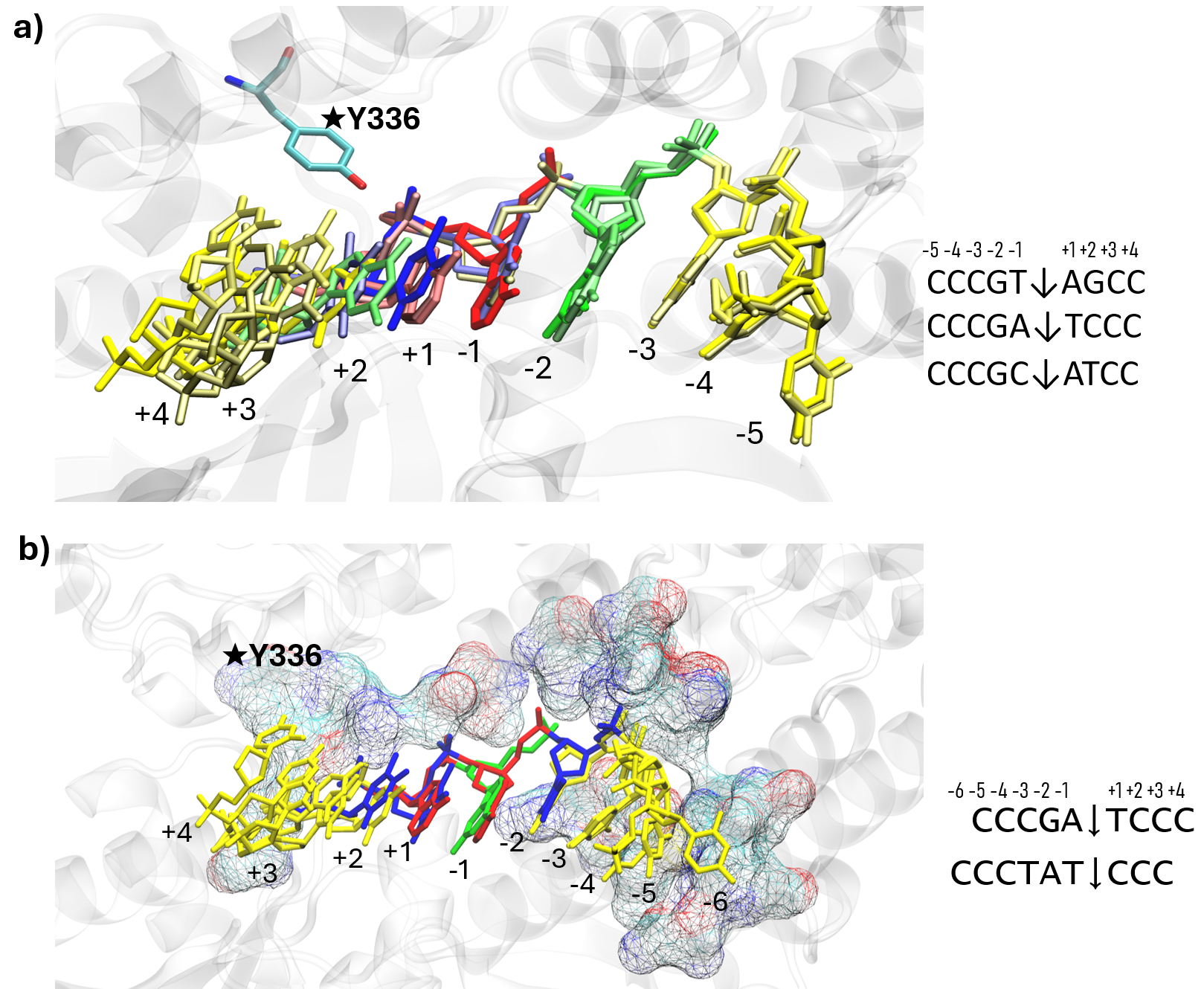


Figure S4. Comparison of substrate placement for sequences starting with a) CCCG- and b) CCCT-. Sequences starting with CCCG were always placed with the first C at –5 position regardless of what bases that follow (a) whereas the sequence starting with CCCT shifts the placement by one base so that the first C placed at –6 position (b). We also highlight the residues lining the substrate in mesh representation.


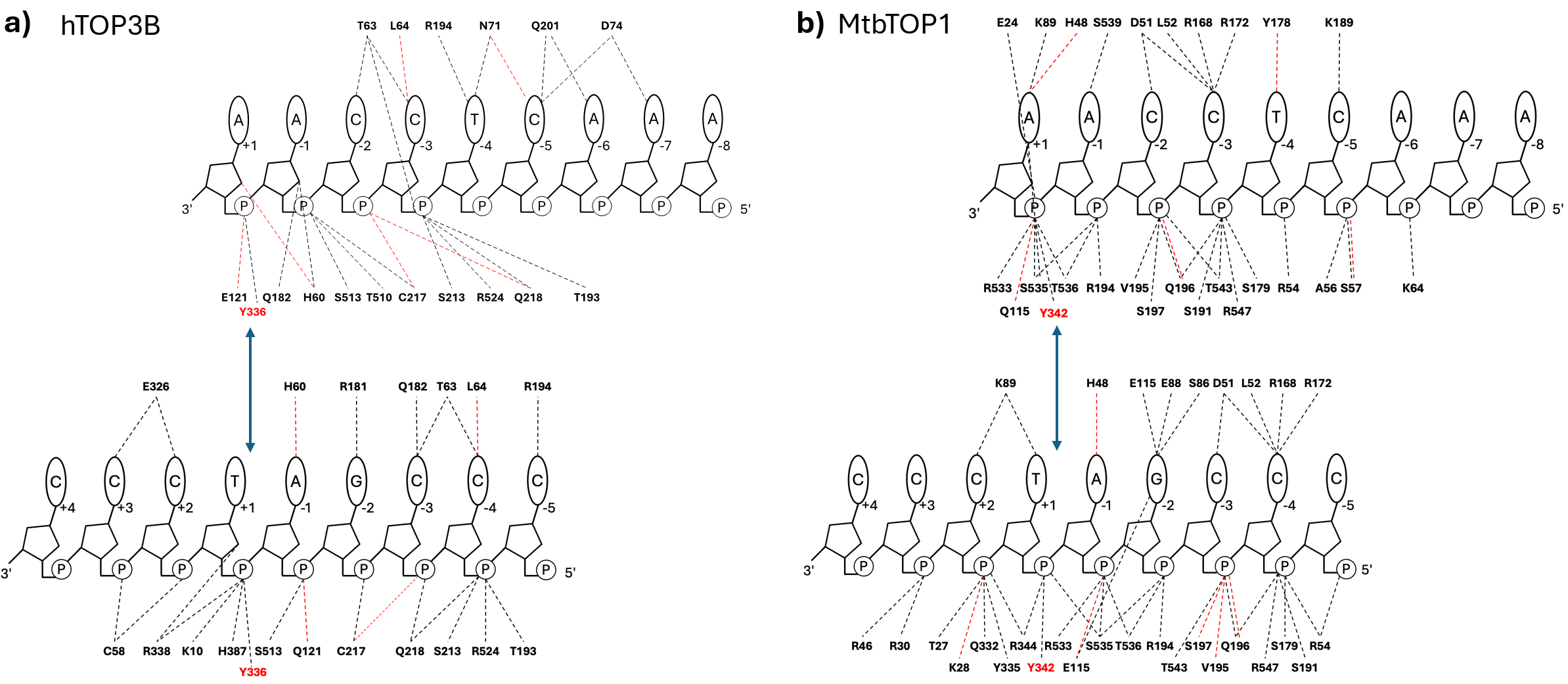


Figure S5. Interaction map for the DNA substrate and protein residues in a) hTOP3B and b) MtbTOP1 for two different sequences, one starting with AAA- and the other starting with CCC-. The position of the catalytic tyrosine (Y336 for hTOP3B and Y342 for MtbTOP1) relative to the sequence placement is shown with an arrow.

Reference:

1. Yang, X.; Saha, S.; Yang, W.; Neuman, K. C.; Pommier, Y., Structural and biochemical basis for DNA and RNA catalysis by human Topoisomerase 3beta. *Nat Commun* **2022,** *13* (1), 4656.
